# Sorting nexin 9 (SNX9) is not essential for development and auditory function in mice

**DOI:** 10.18632/oncotarget.12040

**Published:** 2016-09-15

**Authors:** Chengcheng Liu, Xiaoyan Zhai, Haibo Du, Yujie Cao, Huiren Cao, Yanfei Wang, Xiao Yu, Jiangang Gao, Zhigang Xu

**Affiliations:** ^1^ Shandong Provincial Key Laboratory of Animal Cells and Developmental Biology, School of Life Sciences, Shandong University, Jinan, Shandong 250100, P. R. China; ^2^ Department of Physiology, Shandong University School of Medicine, Jinan, Shandong 250012, P. R. China; ^3^ Current Address: Cell Biology, Department of Biology, Faculty of Science, Utrecht University, CH Utrecht 3584, The Netherlands

**Keywords:** SNX9, knockout mice, inner ear, hearing, hair cells

## Abstract

Sorting nexins are a large family of evolutionarily conserved proteins that play fundamental roles in endocytosis, endosomal sorting and signaling. As an important member of sorting nexin family, sorting nexin 9 (SNX9) has been shown to participate in coordinating actin polymerization with membrane tubulation and vesicle formation. We previously showed that SNX9 is expressed in mouse auditory hair cells and might regulate actin polymerization in those cells. To further examine the physiological role of SNX9, we generated *Snx9* knockout mice using homologous recombination method. Unexpectedly, Snx9 knockout mice have normal viability and fertility, and are morphologically and behaviorally indistinguishable from control mice. Further investigation revealed that the morphology and function of auditory hair cells are not affected by *Snx9* inactivation, and *Snx9* knockout mice have normal hearing threshold. In conclusion, our data revealed that *Snx9*-deficient mice do not show defects in development as well as auditory function, suggesting that SNX9 is not essential for mice development and hearing.

## INTRODUCTION

**Figure 1:**
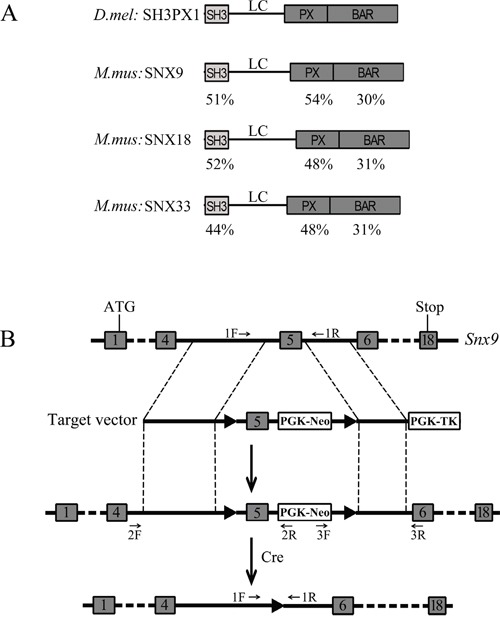
Strategy for generation of *Snx9* knockout mice **A.** Schematic drawing of the domain structures of *Drosophila melanogaster* SH3PX1 and *Mus musculus* SNX9, SNX18 and SNX33. The identity of different domains between SH3PX1 and its mouse homologs is indicated below each domain. **B.** Schematic drawing of targeting strategy for *Snx9* gene disruption. The targeting vector has two *loxP* sites flanking exon 5 of *Snx9* gene. After homologous recombination, the two *loxP* sites are placed flanking exon 5 of *Snx9* gene in the mouse genome. Then exon 5 is excised with the help of Cre recombinase, resulting in disruption of *Snx9* gene. The localizations of genotyping primers in the genome are indicated.

Sorting nexin 9 (SNX9) was initially identified as a Src homology 3 (SH3) domain- and phox homology (PX) domain-containing protein that binds metalloproteinases ADAM9 and ADAM15 [1]. Besides SH3 and PX domains, SNX9 also contains a low complex (LC) region and a Bin-Amphiphysin-Rvs (BAR) domain, all of which are important for SNX9 function (Figure 1A). The N-terminal SH3 domain of SNX9 binds proline rich domain (PRD)-containing proteins, such as dynamins, WASP and N-WASP [2–6]. The LC region is involved in binding Arp2/3 complex as well as AP-2 and clathrin [2, 7]. The C-terminal PX and BAR domains are able to bind multiple phosphoinositides [8–11]. Accordingly, through these domains, SNX9 binds various proteins/lipids that are involved in actin assembly and membrane remodeling. Therefore, it is not surprising that SNX9 has been shown to play important roles in coordinating actin polymerization with membrane tubulation and vesicle formation [12, 13].

SNX9 belongs to sorting nexin protein family whose members are characterized by the presence of a particular type of PX domain – the SNX-PX domain [14]. Sorting nexins are conserved from yeast to mammals. At present, at least 33 mammalian sorting nexins have been identified, most of which were shown to regulate endosomal sorting and/or recycling [15–17]. Among sorting nexin protein family members, SNX9, SNX18 and SNX33 are closely related to each other. They all share characteristic SH3, LC and PX-BAR domains, constituting a separate subfamily [18]. In *Drosophila melanogaster*, the SNX9 subfamily is represented by a single protein, SH3PX1, which shares a similar domain organization as mammalian SNX9/18/33 [19, 20] (Figure 1A). SNX9, SNX18 and SNX33 show different subcellular localizations and appear to function in different trafficking pathways [18]. However, it has been shown that they might be functionally redundant and can compensate for each other during clathrin-mediated endocytosis [21].

Recently we found that SNX9 interacts with F-BAR protein FCHSD1 and might regulate actin polymerization in mouse auditory hair cells [22]. F-BAR proteins possess a FCH-BAR (F-BAR) domain, which is a special BAR domain that binds lipids and induces membrane tubulation [23]. To further elucidate the physiological role of SNX9, we generated *Snx9* knockout mice using homologous recombination method. To our surprise, inactivation of *Snx9* does not affect the development as well as the auditory function in mice.

## RESULTS

### Generation of *Snx9* knockout mice

**Figure 2:**
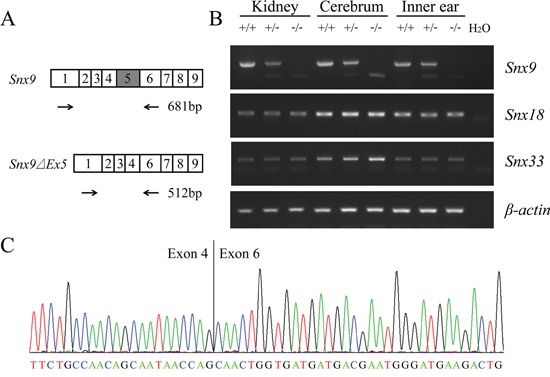
Analysis of *Snx9* knockout mice at mRNA level **A.** Schematic drawing of the strategy for RT-PCR analysis of *Snx9* knockout mice. **B.** RT-PCR was performed to analyze *Snx9* knockout mice. Total RNAs of different tissues were extracted from 1-month-old *Snx9^+/+^*, *Snx9^+/−^* and *Snx9^−/−^* mice. Expression of *Snx9*, *Snx18* and *Snx33* mRNA were examined by performing RT-PCR. *β-actin* was used as an internal control. **C.** The sequence of RT-PCR product from *Snx9^−/−^* mice was determined by sequencing, which confirmed the deletion of exon 5 in *Snx9* transcript.

**Figure 3:**
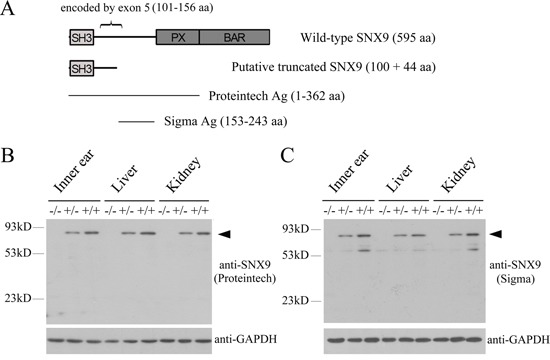
Analysis of *Snx9* knockout mice at protein level **A.** Schematic drawing of the domain architecture of SNX9 as well as the regions recognized by the antibodies used in this study. After deletion of exon 5, a putative truncated SNX9 of 144 amino acids might be expressed. **B.** and **C.** Western blot was performed to analyze *Snx9* knockout mice. Proteins of different tissues were extracted from 3-week-old *Snx9^+/+^*, *Snx9^+/−^* and *Snx9^−/−^* mice, and western blot was performed using specific SNX9 antibodies as indicated. GAPDH was used as internal control. A band with a molecular mass around 90 kDa (indicated by arrowhead), consistent with the expected size of SNX9, was detected with the two anti-SNX9 antibodies in tissues from *Snx9^+/+^* and *Snx9^+/−^* mice, but not in tissues from *Snx9^−/−^* mice.

Mouse *Snx9* gene has 18 exons, coding for 595 amino acids (Figure 1B). In order to inactivate *Snx9*, we decided to delete exon 5, which is 169 bp long. Deletion of exon 5 will cause frameshift and create a premature stop codon, resulting in a putative truncated protein consisting of the N-terminal 100 amino acids and additional 44 unrelated amino acids (Figure 2A and 3A). We constructed a targeting vector, which could create a modified *Snx9* allele carrying two *loxP* sites flanking exon 5 after homologous recombination (Figure 1B). A *neo^R^* cassette was used for G418 selection of ES cells. ES cells with correct recombination were verified by genotyping PCR and Sanger sequencing (Figure S1A and data not shown) and injected into host blastocysts to generate chimeric mice, which were then bred with wild type mice to generate heterozygous *Snx9^lox/+^* mice. Genotyping PCR using genomic DNA as template showed correct recombination in *Snx9^lox/+^* mice (data not shown).

To examine whether *Snx9* is required for mice development, we used *EIIa^Cre/+^* transgenic mice, which mediates efficient and ubiquitous excision of *loxP*-flanked DNA sequences at early stages of development. Mating *loxP*-carrying mice with *EIIa^Cre/+^* mice results in first generation progeny with the *loxP*-flanked sequences deleted from all tissues examined, including the germ cells [24]. *EIIa^Cre/+^* mice were mated with *Snx9^lox/+^* mice to generate *EIIa^Cre/+^*;*Snx9^lox/+^* mice, which were then mated with *Snx9^lox/+^* mice to generate *EIIa^Cre/+^*;*Snx9^lox/lox^* mice. From here on, the exon 5 of *Snx9* was always excised regardless of *EIIa^Cre^* inheritance, hence in the following experiments we designate homozygous and heterozygous *Snx9* mice as *Snx9^−/−^* and *Snx9^+/−^* mice, respectively. The excision of exon 5 in *Snx9^−/−^* and *Snx9^+/−^* mice was confirmed by performing genotyping PCR with genomic DNA and Sanger sequencing (Figure S1B and data not shown).

### Verification of *Snx9* knockout mice

Next we performed RT-PCR to examine whether transcription of *Snx9* was affected in *Snx9* knockout mice. Total RNAs extracted from various tissues of different mouse genotypes were reverse transcribed into cDNA and amplified using primers flanking the deleted region (Figure 2A). A large fragment corresponding to exon 5 inclusion was amplified from wild type mice. In contrast, a small fragment corresponding to exon 5 excision was amplified from homozygous *Snx9^−/−^* mice. Both fragments were amplified from heterozygous *Snx9^+/−^* mice (Figure 2B). The abundance of the small fragment is significantly lower than that of the large fragment, which is possibly caused by nonsense-mediated mRNA decay (NMD) [25]. The amplified fragments were sequenced and the excision of exon 5 sequence in *Snx9* mRNA from *Snx9^−/−^* and *Snx9^+/−^* mice were confirmed (Figure 2C). We also amplified the full-length *Snx9* cDNA from *Snx9^−/−^* mice and wild type mice by performing RT-PCR, and confirmed that the deletion of exon 5 indeed causes the expected frameshift in *Snx9^−/−^* mice by Sanger sequencing (data not shown).

We then examined whether SNX9 protein expression was affected in *Snx9* knockout mice by performing western blot analysis. Proteins from various tissues of different mouse genotypes were separated in SDS-PAGE. SNX9 expression was examined using a polyclonal anti-SNX9 antibody that recognizes the N-terminal 362 amino acids of SNX9 (Figure 3A). A specific band with a molecular mass around 90 kDa, consistent with the expected size of SNX9, was detected in tissues from *Snx9^+/+^* and *Snx9^+/−^* mice. Meanwhile, this band was not detectable in tissues from *Snx9^−/−^* mice, suggesting that the expression of SNX9 protein was disrupted in *Snx9^−/−^* mice (Figure 3B). We also performed western blot analysis using a different polyclonal antibody that recognizes amino acids 153-243 of SNX9 (Figure 3A). Multiple bands were detected with this antibody, many of which were not detected with the first anti-SNX9 antibody, suggesting that the second antibody is multi-specific (Fic. 3C). Nevertheless, a band with a molecular mass around 90 kDa was detected in tissues from *Snx9^+/+^* and *Snx9^+/−^* mice, but not in tissues from *Snx9^−/−^* mice, confirming that the expression of SNX9 protein was indeed disrupted in *Snx9^−/−^* mice (Figure 3C). Based on these data, we conclude that *Snx9^−/−^* mice lack functional SNX9 protein.

### Development of *Snx9* knockout mice is normal

SNX9 has been suggested to play fundamental roles in endocytosis regulation, so we expected that *Snx9* knockout mice might show developmental deficits. Unexpectedly, *Snx9^−/−^* mice are morphologically and behaviorally indistinguishable from *Snx9^+/−^* or wild type mice. Interbreeding of *Snx9^+/−^* mice gave rise to offspring in the expected Mendelian ratio (22.7% wild-type, 47.7% *Snx9^+/−^*, and 29.5% *Snx9^−/−^*; n=44; χ^2^ test P value=0.78) that have normal viability.

**Figure 4:**
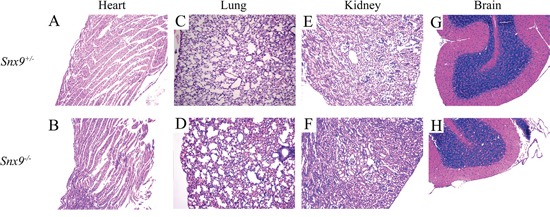
*Snx9* knockout mice do not display pathological changes Tissues of 2-month-old *Snx9^+/−^* and *Snx9^−/−^* mice were sectioned and stained with hematoxylin-eosin (HA). Shown are results from heart **A, B.** lung **C, D.** kidney **E, F.** and brain **G, H.**

SNX9 is ubiquitously expressed in multiple tissues in mice, including heart, lung, kidney, brain, pancreas, spleen, et al [1]. We then performed hematoxylin-eosin (HE) staining of the tissues exhibiting abundant SNX9 expression to look for potential pathological changes. Histological analysis of heart, lung, kidney and brain from adult *Snx9^−/−^* mice revealed no abnormalities (Figure 4A-4H). Furthermore, male and female *Snx9^−/−^* mice were fertile, indicating no defects in reproductive functions or germ cell production.

### Auditory function of *Snx9* knockout mice is normal

**Figure 5:**
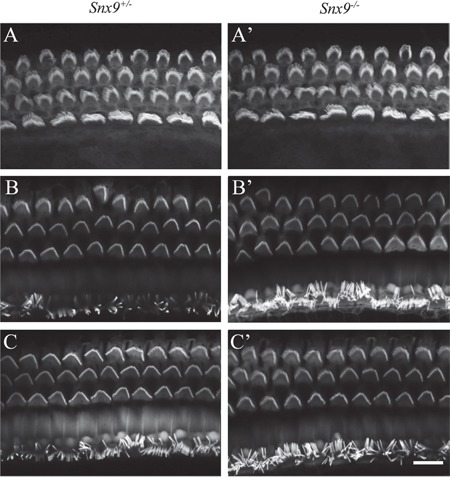
Auditory hair cell stereocilia are morphologically normal in *Snx9* knockout mice Auditory hair cell stereocilia of *Snx9^+/−^* and *Snx9^−/−^* mice were stained with TRITC-conjugated phalloidin and imaged using confocal microscope. Images were taken from the middle turn of the cochlea. **(A)** and **(A')** Postnatal 8 days. **(B)** and **(B')** Postnatal 4 weeks. **(C)** and **(C')** Postnatal 8 weeks. Scale bar, 10 μm.

**Figure 6:**
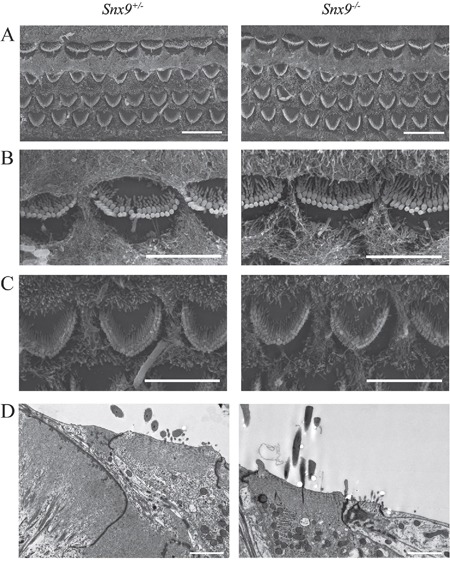
Auditory hair cell stereocilia are morphologically normal in *Snx9* knockout mice **A.** Low-magnification SEM images of cochlear stereociliary bundles of *Snx9^+/−^* and *Snx9^−/−^* mice. **B.** High-magnification SEM images of inner hair cell stereociliary bundles of *Snx9^+/−^* and *Snx9^−/−^* mice. **C.** High-magnification SEM images of outer hair cell stereociliary bundles of *Snx9^+/−^* and *Snx9^−/−^* mice. Images were taken from the mid-apical turn of postnatal day 7 mice. **D.** High-magnification TEM images of inner hair cells of 2-month-old *Snx9^+/−^* and *Snx9^−/−^* mice. Scale bars, 10 μm in (A), 5 μm in (B) and (C), 2 μm in (D).

Confocal microscopy of phalloidin-stained whole-mounts was employed to examine the morphology of cochlear hair cell stereocilia. The result did not reveal any abnormality in *Snx9^−/−^* mice at postnatal 8-day, 4-week and 8-week (Figure 5A-5C'). To examine the morphology of cochlear hair cell stereocilia at higher resolution, we performed scanning electron microscopy (SEM) and transmission electron microscopy (TEM), which also did not reveal any difference between *Snx9* knockout mice and control mice (Figure 6A-6D). These results suggest that loss of *Snx9* does not affect the morphology of cochlear hair cell stereocilia.

**Figure 7:**
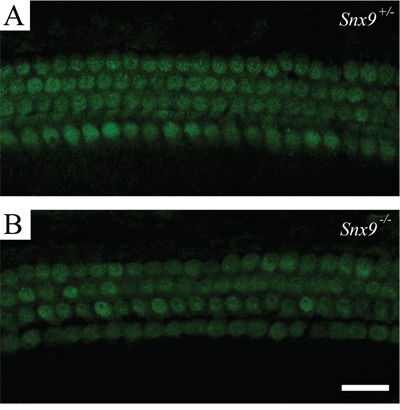
Hair cells of *Snx9* knockout mice are functionally normal FM1-43FX uptake by auditory hair cells of postnatal day 8 *Snx9^+/−^* **(A)** and *Snx9^−/−^* **(B)** mice were examined using confocal microscope. Scale bar, 20 μm.

**Figure 8:**
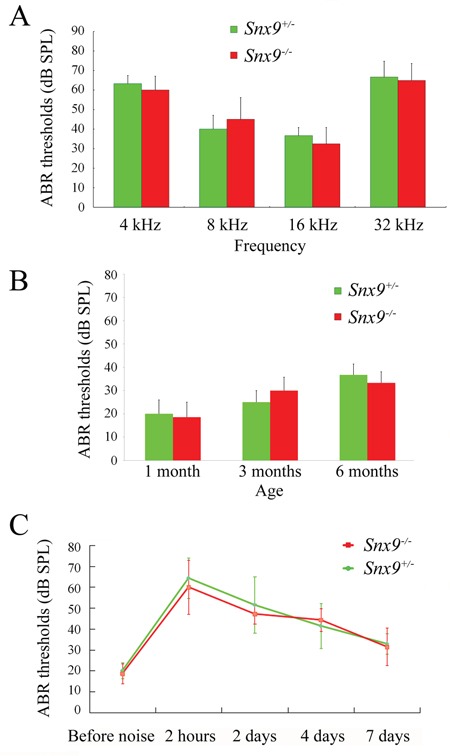
Auditory brainstem response (ABR) measurements show normal auditory function of *Snx9* knockout mice **A.** ABR thresholds of 1-month-old *Snx9^+/−^* (n=3) and *Snx9^−/−^* (n=4) mice to 4, 8, 16 and 32 kHz stimuli were measured. **B.** ABR thresholds of 1-month-old (n=9 for each genotypes), 3-month-old (n=6 for each genotypes), and 6-month-old (n=3 for each genotypes) *Snx9^+/−^* and *Snx9^−/−^* mice to click stimuli were measured. **C.** 1-month-old *Snx9^+/−^* (n=7) and *Snx9^−/−^* (n=7) mice were subjected to 8 kHz noise at 106 dB DSL for 90 minutes, and ABR thresholds were measured pre-exposure and at different post-exposure time points as indicated. No significant differences were observed between genotypes. Variance bars indicate standard error.

Florescent dye FM1-43 labels hair cells by transporting through the mechanosensitive channels, thus provides an index of the functional integrity of hair cells [26, 27]. Here we used FM1-43FX, a fixable analog of FM1-43, to examine the function of cochlear hair cells. FM1-43FX labeling in *Snx9^−/−^* hair cells is indistinguishable from *Snx9^+/−^* hair cells, suggesting that hair cell function is not affected in *Snx9^−/−^* mice (Figure 7). Auditory brainstem response (ABR) measurements were then performed to evaluate the auditory function of *Snx9* knockout mice. No significant differences in ABR thresholds were found between 1-month-old *Snx9^−/−^* and *Snx9^+/−^* mice in all frequencies examined (Figure 8A). Click-evoked ABR measurement also did not reveal any significant difference between *Snx9^−/−^* and *Snx9^+/−^* mice at age of 1 month, 3 months, and 6 months (Figure 8B).

To investigate whether *Snx9* knockout mice show increased acoustic vulnerability, we exposed *Snx9^−/−^* and *Snx9^+/−^* mice to 8 kHz noise at 106 dB SPL for 90 minutes. We compared ABR thresholds of both genotypes before and after the noise exposure, and did not detect any significant difference between *Snx9^−/−^* and *Snx9^+/−^* mice (Figure 8C). Taken together, our results suggest that the auditory function of *Snx9* knockout mice is normal.

## DISCUSSION

Sorting nexins are a large family of evolutionarily conserved proteins that play fundamental roles in endocytosis, endosomal sorting and signaling. RNA sequencing results of FACS-sorted cells (SHIELD; https://shield.hms.harvard.edu) revealed that many sorting nexins are expressed in the mouse inner ear [28, 29]. As an important member of sorting nexin protein family, SNX9 coordinates actin polymerization with membrane tubulation and vesicle formation, and plays important roles in endocytosis [12, 13]. SNX9 negatively regulates invadopodia formation, and its expression is lowered in primary tumors [30]. Meanwhile, SNX9 expression is increased in metastases, and could promote metastasis by enhancing cancer cell invasion [31]. These results suggest a complex role of SNX9 in cancer cells. At present, the functional role of SNX9 in a physiological context remains elusive.

In order to examine the physiological function of SNX9, we generated *Snx9* knockout mice by deleting exon 5 of *Snx9* gene using homologous recombination method. Exon 5 of *Snx9* gene is 169 bp long, encoding amino acids 101-156 of SNX9 protein. After deletion of exon 5, exon 4 is spliced to exon 6, which causes frameshift mutation and might leave a putative truncated SNX9 protein of 144 amino acids. We couldn't detect this truncated protein in *Snx9* knockout mice, possibly because it is unstable, or it's too small to be detected in western blot. Nevertheless, given the fact that this putative truncated SNX9 only contains the SH3 domain, it will be nonfunctional even it exists in knockout mice.

We confirmed at different levels that exon 5 deletion resulted in *Snx9* deficiency. At the genome level, deletion of exon 5 was confirmed by genotyping PCR and Sanger sequencing (Figure S1 and data not shown). At the mRNA level, deletion of 169 bp and the resulted frameshift in *Snx9* ORF were confirmed by RT-PCR and Sanger sequencing (Figure 2). Significant reduction of *Snx9* mRNA in *Snx9* knockout mice was also observed, which is probably caused by nonsense-mediated mRNA decay (NMD) (Figure 2). At the protein level, disruption of SNX9 protein expression was confirmed by western blot analysis using two different polyclonal anti-SNX9 antibodies (Figure 3).

Our data showed that *Snx9* deficiency does not affect the development in mice. Histological analysis of heart, lung, kidney and brain from adult *Snx9^−/−^* mice revealed no abnormalities (Figure 4). *Snx9^−/−^* mice are morphologically and behaviorally indistinguishable from *Snx9^+/−^* or wild type mice, and have normal viability and fertility. These results suggest that development is not affected in *Snx9* deficient mice.

We then examined the auditory function of *Snx9* deficient mice in details. The morphology of auditory hair cell bundles was examined using confocal microscopy, SEM, and TEM, and no difference was detected between control and knockout mice (Figure 5 and 6). FM1-43FX uptake experiment was performed to evaluate the function of auditory hair cells, and once again, no difference was revealed between control and knockout mice (Figure 7). Finally, ABR measurement was employed to evaluate the auditory function of mice under normal and noise conditions, and the results showed that there is no difference between control and knockout mice (Figure 8). Taken together, our results suggest that the auditory function of *Snx9* knockout mice is normal, although more subtle defects might exist that were not evaluated in the present work.

Considering the important role of SNX9 in coordinating actin polymerization with membrane tubulation and vesicle formation, it is quite unexpected that loss of *Snx9* does not affect development and auditory function in mice. The lack of abnormalities in *Snx9* knockout mice suggests that SNX9 is not necessary for mice development and auditory function, or alternatively, *Snx9* deficiency could be compensated by another protein(s). As mentioned above, SNX18 and SNX33 are paralogs of SNX9, which share SH3, LC and PX-BAR domains and form a separate sorting nexin subfamily [18]. SNX9/18/33 are ubiquitously expressed in various tissues and cell types, and all of them are involved in endocytosis regulation [21, 32]. More than that, it has been shown that SNX9 and SNX18 are functionally redundant and can compensate for each other in endocytosis [21]. Hence we speculate that SNX18 and/or SNX33 might compensate for the loss of SNX9 in *Snx9* knockout mice. RT-PCR was then performed to examine the expression of *Snx18* and *Snx33* in different tissues from *Snx9^+/+^*, *Snx9^+/−^* and *Snx9^−/−^* mice. The expression of *Snx18* and *Snx33* was detected in all genotypes, although no obvious upregulation in *Snx9^−/−^* mice was found, consistent with the possibility that they could compensate for the loss of *Snx9* (Figure 2B).

In line with this, a recent work showed that SNX9, SNX18, and SNX33 all bind to FCHSD1. Moreover, inactivation of *Sh3px1*, the only homolog of *Snx9/18/33* in *Drosophila*, caused neurotransmitter release deficiency, but *Sh3px1*null flies were still viable [20]. Compared with mice, the fly genome contains a single *Snx9/18/33* homolog, *Sh3px1*, which makes the loss-of-function analysis much easier. It remains unknown whether there is hearing deficit in *Sh3px1*null flies. If the auditory function of *Sh3px1*null flies is normal, it might suggest that none of SNX9, SNX18, and SNX33 is required for hearing in mice. In the future, triple knockout mice may help to fully understand the physiological function of SNX9 as well as SNX18 and 33.

## MATERIALS AND METHODS

### Ethics statement

All animal experiments were approved by the Ethics Committee of Shandong University (Permit Number: ECAESDUSM 20123004) and conducted in accordance with the approved guidelines.

### Generation of *Snx9* knockout mice

*Snx9^loxP/+^* mice were generated by Shanghai Biomodel Organism Science & Technology Development Co., Ltd. A targeting vector was constructed to allow insertion of two *loxP* sites flanking exon 5 of *Snx9* gene. A neomycin resistant gene and an HSV-thymidine kinase gene driven by the PGK promoter were included for positive and negative selection, respectively. Following electroporation of the targeting vector into embryonic stem cells (ESCs), G418 and ganciclovir double-resistant colonies were selected and screened by PCR. Homologous recombinant ESCs were microinjected into C57BL/6 female mice to obtain heterozygous *Snx9^loxP/+^* mice, which were then crossed with EIIa-Cre transgenic mice to eventually obtain *Snx9^−/−^* mice. The sequences of genotyping PCR primers are as follows. 1F:5′- ACCTAAGTAGAAGAACCTACTGTA-3′; 1R:5′- AACAGTGTCTCCGTTAGTGAGCAA-3′; 2F: 5′-TGGCTGGACGATAGTCTCCCTCCAT-3′; 2R: 5′-CTGAGCCCAGAAAGCGAAGGA-3′; 3F: 5′-CCTCCCCCGTGCCTTCCTTGAC-3′; 3R: 5′-CTTCATCCCATTCGTCATCATCACC-3′.

### RNA extraction and RT-PCR

Total RNA from different mouse tissues or mouse embryonic fibroblast (MEF) cells was extracted using RNeasy Micro Kits (Qiagen, Valencia, CA) according to the manufacturer's protocol. Reverse transcription (RT) was carried out at 42°C for 1 hour in a 20μL reaction mixture containing 1μg of total RNA, 10 pmol of oligo-dT, and 200 units of Super-Script III reverse transcriptase (Invitrogen, Carlsbad, CA). Polymerase chain reaction (PCR) was performed using the cDNA as template with the following primers: *Snx9* forward primer, 5′-AGTCGACCTCCCCGCCCGCTTAG-3′, *Snx9* reverse primer, 5′-aggaggcacccgcacgactgtttc-3′ (681bp), *Snx18* forward primer, 5′-ggccaacttcccagacatcat-3′, *Snx18* reverse primer, 5′-caacgtggcgaaggaaatagtg-3′ (148bp), *Snx33* forward primer, 5′-atgaaaccgttggcgaaatg-3′, *Snx33* reverse primer, 5′-catatgcttaaaatcgagct-3′ (286bp), *β-actin* forward primer, 5′-ACGGCCAGGTCATCACTATTG-3′, *β-actin* reverse primer, 5′-AGGGGCCGGACTCATCGTA-3′ (372bp).

### Western blot

Mouse tissues were dissected and homogenized in ice-cold lysis buffer consisting of 150 mM NaCl, 50 mM Tris at pH 7.5, 1% NP-40, 0.1% SDS, 1% (vol/vol) Triton X-100, 1 mM PMSF, and 1 X protease inhibitor cocktail (Sigma-Aldrich, Saint Louis, MO). After centrifugation at 4°C, the supernatant was collected and separated by polyacrylamide gel electrophoresis (PAGE), then transferred to PVDF membrane. After blocking in PBS containing 5% BSA and 0.1% Tween-20, the membrane was incubated with rabbit anti-SNX9 polyclonal antibody (Proteintech, Cat. No. 15721-1-AP, or Sigma-Aldrich, Cat. No. HPA031410) at 4°C over night, followed by incubation with HRP-conjugated goat anti-rabbit secondary antibody (Bio-Rad, Cat. No. 170-6515) at 4°C for an hour. The signals were detected with the ECL system (Cell Signaling Technology, Danvers, MA). Then the same blot was incubated with mouse anti-GAPDH polyclonal antibody (Millipore, Cat. No. MAB374) at 4°C over night, followed by incubation with HRP-conjugated goat anti-mouse secondary antibody (Bio-Rad, Cat. No. 170-6516) at 4°C for an hour. The signals were detected with the ECL system (Cell Signaling Technology).

### Histology

For histological staining, mouse tissues were fixed in 4% paraformaldehyde overnight at 4°C, then dehydrated and embedded in paraffin. Sections (3 μm) were deparaffinized, rehydrated, and histologically stained with hematoxylin-eosin (HE) according to standard protocols, then imaged with a light microscope (Nikon YS100, Japan).

### Phalloidin staining of hair cell stereocilia

Mouse basilar membrane was dissected and fixed with 4% paraformaldehyde, then permeabilized with PBT1 buffer (0.1% Triton X-100, 1% BSA, 5% heat-inactivated donkey serum in PBS, pH 7.3) for 30 minutes. The samples were then incubated with 0.4 μg/ml TRITC-conjugated phalloidin (Sigma) in PBT2 (0.1% Triton X-100, 0.1% BSA) for 30 minutes followed by three 10-minutes washes with PBT2, then mounted in PBS-glycerol (1:1) and imaged with a confocal microscope (LSM 700, Zeiss, Germany). All steps were performed at room temperature.

### Scanning Electron Microscopy (SEM)

Mouse inner ears were dissected and fixed with 2.5% glutaraldehyde in 0.1 M phosphate buffer overnight at 4°C. Cochleae were dissected out of the temporal bone, post-fixed with 1% osmium tetroxide in 0.1 M phosphate buffer, followed by dehydration in ethanol and critically point drying. Samples were then mounted and sputter coated with gold, and imaged using a Quanta250 Field-Emission scanning electron microscope (FEI, The Netherlands).

### Transmission Electron Microscopy (TEM)

Mouse inner ears were dissected and fixed with 2.5% glutaraldehyde in 0.1 M phosphate buffer overnight at 4°C. The specimens were post-fixed for 1.5 h with 1% osmium tetroxide in phosphate buffer, then dehydrated through a graded ethanol series and embedded in Epon812. Ultrathin sections (70 nm) were prepared, stained with uranyl acetate and lead citrate, and examined by electron microscopy (JEOL-1200EX).

### FM1-43FX dye uptake assay

FM1-43FX (Molecular Probes, Invitrogen), a fixable analog of FM1-43 [N-(3-triethylammoniumpropyl)-4-(4-(dibutylamino)-styryl) pyridinium dibromide] was used to label functional mechanotransducing hair cells. Mouse basilar membrane was dissected and placed in PBS containing 3 μM FM1-43FX for 30 seconds and rinsed three times in PBS, then fixed with 4% paraformaldehyde at room temperature for 20 minutes. After washing three times with PBS, the samples were mounted in PBS-glycerol (1:1) and imaged with a confocal microscope (LSM 700, Zeiss, Germany).

### Auditory brainstem response (ABR) measurements

Mice were anesthetized intraperitoneally with 8.4 mg/100g Pentobarbital. Body temperature was maintained at 37 °C by placing the mice on an isothermal pad during testing and recovery from anesthesia. Electrodes were inserted subcutaneously at the vertex and pinna, and a ground electrode was placed near the tail. The stimulus generation, presentation, ABR acquisition and data management were coordinated using RZ6 workstation and BioSig software (Tucker Davis Technologies, Inc.). Specific acoustic stimuli (clicks or tone bursts of 4, 8, 16 and 32 kHz) were generated using high frequency transducers. At each sound level, 512 responses were sampled and averaged. ABR thresholds were obtained for each animal by reducing the stimulus intensity in 10 dB SPL steps from 90 dB SPL to identify the lowest intensity at which all ABR waves were detectable.

### Noise exposure

Mice were located in a sound-exposure chamber with loudspeakers driven by a power amplifier (AWA5870B; Aihua Instruments Co., Hangzhou, China). Audio sound files were created and equalized with audio editing software (AWA6290M; Aihua Instruments Co.). Sound levels were calibrated with a sound level meter (AWA6221A; Aihua Instruments Co.) at multiple locations within the chamber. Mice of different genotypes were exposed to 8 kHz noise at 106 dB SPL (sound pressure level) for 90 minutes, and then put back in their home cage. ABR thresholds were measured pre-exposure and at different post-exposure time points.

### Statistical analysis

Data were shown as mean ± standard error. Student's t test was used for statistical analysis. P < 0.05 was considered statistically significant.

## SUPPLEMENTARY MATERIALS FIGURE


