# Supplementary material for: Sorting nexin 9 (SNX9) is not essential for development and auditory function in mice
Source: Oncotarget. 2016 Sep 15;7(42):68921–32. doi: 10.18632/oncotarget.12040 (PMC5356600; doi:10.18632/oncotarget.12040)
Supplement: Supplementary file 1 [file oncotarget-07-68921-s001.pdf]

Sorting nexin 9 (SNX9) is not essential for development and auditory function in mice

SUPPLEMENTARY FIGURE

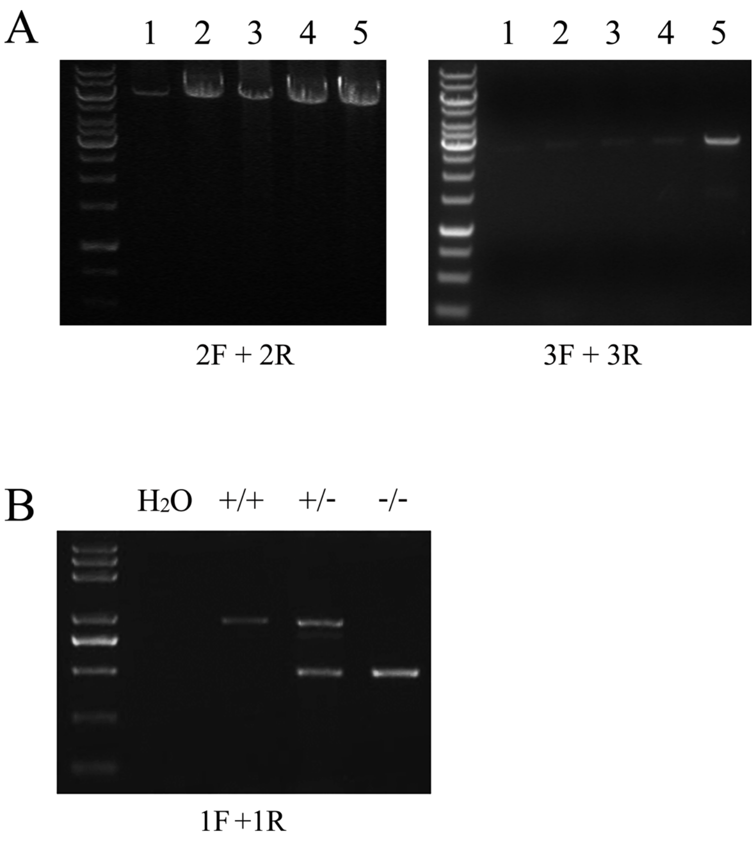

Supplementary Figure 1: Genotyping analysis of *Snx9* knockout mice. A. Genotyping analysis of embryonic stem (ES) cells after G418 and ganciclovir selection. A faint band was amplified from negative control (lane 1, ES cells without recombination), which likely results from unspecific binding of the primers. Nevertheless, a much stronger band with the expected molecular mass was obtained from the positive ES clones (lane 2-5). **B.** Genotyping analysis of *Snx9* knockout mice.
